# High-performance spinel NiMn_2_O_4_ supported carbon felt for effective electrochemical conversion of ethylene glycol and hydrogen evolution applications

**DOI:** 10.1038/s41598-023-50950-3

**Published:** 2024-01-04

**Authors:** Shymaa S. Medany, Mahmoud A. Hefnawy, Soha M. Kamal

**Affiliations:** 1 Department of Chemistry, Faculty of Science, Cairo University, Giza, 12613 Egypt; 2 Applied Electrochemistry Laboratory, Chemistry Department, Faculty of Science, Beni-Suef University, Beni-Suef, 52511 Egypt

**Keywords:** Chemistry, Catalysis, Electrochemistry, Energy, Environmental chemistry, Medicinal chemistry

## Abstract

One of the most effective electrocatalysts for electrochemical oxidation reactions is NiMn_2_O_4_ spinel oxide. Here, a 3-D porous substrate with good conductivity called carbon felt (CF) is utilized. The composite of NiMn_2_O_4_-supported carbon felt was prepared using the facile hydrothermal method. The prepared electrode was characterized by various surface and bulk analyses like powder X-ray diffraction, X-ray photon spectroscopy (XPS), Scanning and transmitted electron microscopy, thermal analysis (DTA), energy dispersive X-ray (EDX), and Brunauer–Emmett–Teller (BET). The activity of NiMn_2_O_4_ toward the electrochemical conversion of ethylene glycol at a wide range of concentrations was investigated. The electrode showed a current density of 24 mA cm^−2^ at a potential of 0.5 V (vs. Ag/AgCl). Furthermore, the ability of the electrode toward hydrogen evaluation in an alkaline medium was performed. Thus, the electrode achieved a current density equal 10 mA cm^−2^ at an overpotential of 210 mV (vs. RHE), and the provided Tafel slope was 98 mV dec^−1^.

## Introduction

The escalating global need for energy has been the catalyst for the advancement of sustainable and environmentally conscious alternative energy sources that have the potential to supplant conventional fossil fuels. The utilization of fossil fuels is linked to various constraints, such as the emission of greenhouse gases and the finite nature of these resources. Researchers have also directed their attention toward other intelligent alternatives^1–4^.

Alkaline direct alcohol fuel cells (ADAFCs) powered by compact organic molecules have exhibited potential as portable energy solutions^5,6^. In comparison to traditional low-temperature hydrogen fuel cells, liquid fuel cells have several advantages. Its advantages include increased density and theoretical energy efficiency, enhanced safety measures, and increased transportability and storage convenience. Ethylene glycol (EG) possesses significant promise as a feasible fuel alternative for use in ADAFCs, owing to its enhanced energy density and boiling point compared to methanol and ethanol. Furthermore, empirical evidence suggests that ethylene glycol’s oxidation rate is greater in an alkaline medium than in an acidic medium. A wide variety of catalyst materials can be utilized in alkaline settings, demonstrating significant catalytic efficiency while still being reasonably cost-effective. In an alkaline environment, ethylene glycol undergoes complete oxidation, resulting in the liberation of a total of 10 electrons per molecule, as illustrated below^7^:

1 $$\left( {{\text{CH}}_{{2}} {\text{OH}}} \right)_{{2}} + {10}OH^{ - } \leftrightarrow {\text{ 2 CO}}_{{2}} + {\text{8 H}}_{{2}} {\text{O}} + {1}0e^{ - }$$

Xin et al.^8^ recommended two reaction paths for ethylene glycol (EG) conversion in their study. These pathways involve the creation of oxalate via glycolate oxidation and the cleavage of the carbon–carbon (CC) bond in EG, resulting in the reproduction of species and the ultimate formation of carbonate. In contrast to previous research, Miyazaki et al.^9^ proposed a unified pathway consisting of a series of chemical steps: EG → glycolate → oxalate → CO_2_ → carbonate. The production of glycolate and carbonate appeared to occur via a common intermediate, whereas oxalate appeared to be generated by the oxidation of desorbed glycolate^10,11^.

Nevertheless, the successful integration of EG fuel cells into the commercial sector necessitates the availability of anode catalysts for ethylene glycol oxidation reaction (EGOR) that exhibit exceptional efficiency and cost-effectiveness. Extensive research has been conducted on EGOR over a considerable period of time, with a specific focus on its conduct on various metal and modified metal electrodes. The electrodes utilized in this study consist of platinum (Pt), palladium (Pd), gold (Au), cobalt (Co), iron (Fe), and nickel (Ni). Previous research on these electrodes has predominantly focused on experiments conducted in alkaline environments^12–18^. Pt and Pt-based catalyst anodes play a crucial role due to their high efficiency towards the electrochemical oxidation of the EG and the ability to facilitate reactions at lower potential values. Nevertheless, it should be noted that the cost of these materials is very high and they are susceptible to contamination by CO-adsorbed species^19,20^. Nickel-based materials have garnered considerable interest in the field of electrocatalysis due to their remarkable catalytic capabilities in facilitating the reactions of diverse small organic molecules. These materials encompass metallic nickel, nickel oxides, hydroxides and oxyhydroxides, as well as nickel alloys and composites. Therefore, these materials have been widely utilized as anodic catalysts for electrooxidation processes in recent decades^21–23^.

A technique known as the hydrogen evolution reaction (HER) uses an electric current to turn water into hydrogen gas. Fuel cells, electricity generation, and chemical synthesis are just a few uses for hydrogen, a clean and renewable energy source^24,25^. Hydrogen evolution reactions (HER) play a vital role in reducing The reliance on fossil fuels, which serve as a notable contributor to atmospheric pollution and the release of greenhouse gases. The primary objective of the HER is to address and alleviate the adverse impacts that arise from the extraction and transportation of fossil fuels on both ecosystems and human health. Furthermore, the utilization of high-efficiency rectifiers (HER) facilitates the smooth incorporation of sustainable energy sources, such as solar and wind power, into the preexisting energy framework. Due to their intermittent and variable characteristics, renewable energy sources pose challenges for grid stability and storage.

Carbon felt (CF) as support was used extensively in electrocatalysis field^26–30^. It was an attractive support to the scientists due to its properties for this different metal forms were loaded on its surface. *Pierożyński *et al*.* prepared ruthenium-modified nickel-coated carbon fiber and studied its performance as an electrocatalyst for hydrogen evolution reaction^26^. Koca et al. modified the carbon felt by NiGa for hydrogen production purposes. The authors proved that the presence of Ga on Ni/carbon felt reduces the hydrogen evolution potential of the electrode and provide higher current passages^27^. Ece Altunbaş Şahin was prepared AgCo/CF, using an electrochemical deposition process, a little amount of Ag was deposited over Co-deposited Carbon felt and examined for HER performances in an alkaline medium^31^. The authors proved that the binary AgCo/CF has a high current density of 159.71, 574.10, and 1640.2 mA.g.C at different potentials obtained from cathodic current potential curves of *i*_-1.2V_, *i*_-1.3V_, and *i*_-1.4V_; respectively^31^. Manganese-based materials have received a great attention from researchers toward HER, and/or OER, i.e. water splitting^32^. Fe doped Ni_3_S_2_/MnS was found to have a high catalytic activity towards OER^33^. The combination of heterojunction, which enhances the catalyst's conductivity, modifies the active site's electrical configuration, and maximizes the adsorption capacity of intermediates containing oxygen to produce quick reaction kinetics and excellent catalytic performance, is responsible for the increased catalytic activity^33^. Zhu et al. prepared Se-MnS/NiS heterojunctions as highly efficient bifunctional electrocatalysts for overall water splitting^34^. The electrocatalysis of HER and OER may benefit from the addition of the Se dopant, which could modify the structure and raise the electrochemically active surface area. Furthermore, in comparison to the NiSe, NiS, and Se-NiS catalysts, the synergistic impact of the Se-MnS/NiS heterojunctions encouraged the adsorption of hydrogen atoms on the catalyst's surface. The resulting Se-NiS/MnS catalyst was able to provide a 10 mA cm^−2^ current density for HER and OER in alkaline media, respectively, with overpotentials as low as 56 mV and 211 mV. Additionally, Se-MnS/NiS demonstrated exceptional endurance for 48 h and a comparatively low voltage of 1.47 V at 10 mA cm^−2^ when used directly as bifunctional electrodes for total water splitting^34^.

For the previous reasons, we aimed in our study to synthesize nickel manganese spinel oxide supported on carbon felt, via the hydrothermal method, as electrocatalyst for EGOR and hydrogen production. In an alkaline medium, the activity of modified NMO-CF for ethylene glycol electrooxidation and hydrogen production was investigated. The surface characterizations of the prepared electrocatalyst were studied using several surface techniques. Different electrochemistry techniques were utilized to determine the activity of the modified electrode.

## Experimental section

### Preparation of NiMn_2_O_4_-CF (NMO-CF)

**Figure 1:**
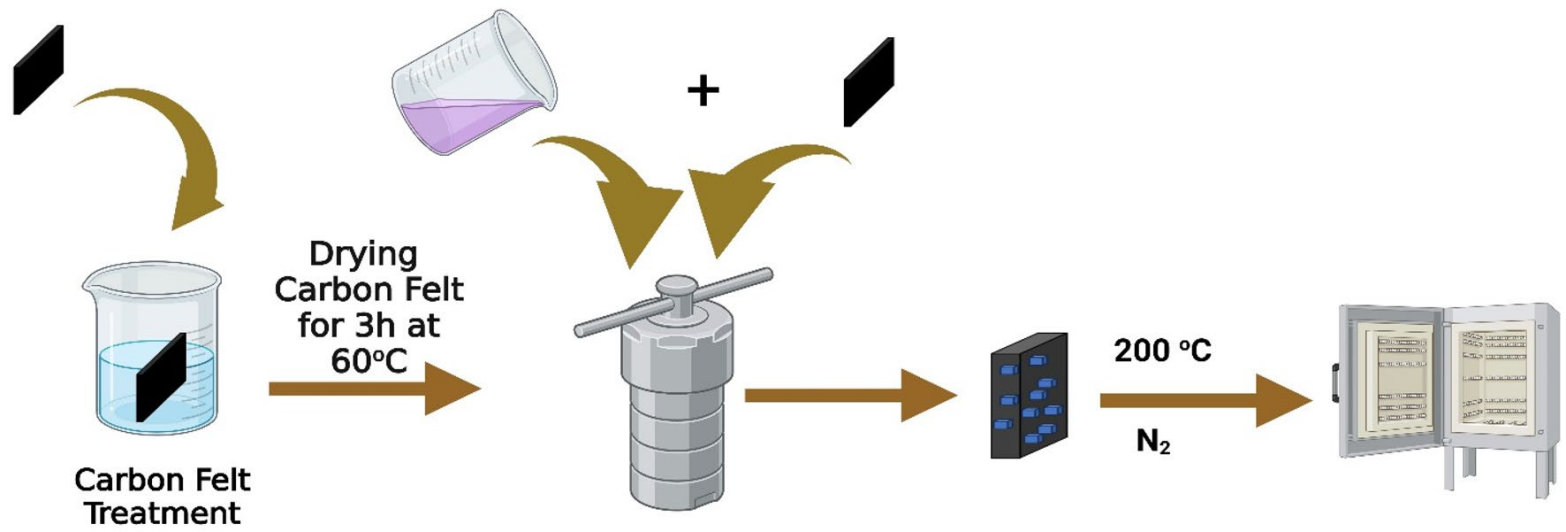
Schematic representation of NMO-CF preparation.

All the chemical reagents utilized in this study were of analytical grade and were not subjected to additional processing. The synthetic pathway is depicted in Fig. 1. To improve the wettability of the carbon felt (CF), a treatment was conducted using a mixture of H_2_SO_4_ and H_2_O_2_ in a volume ratio 1:3. The treatment duration was 3 h at a temperature of 80 °C. Subsequently, CF was thoroughly rinsed with deionized water. Then, the sample was subjected to sonication in ethanol for 30 min, followed by a drying period of 3 h at a temperature of 80 °C^35,36^.

Furthermore, the objective of this procedure was to eliminate the impurities and enhance the surface roughness of carbon fiber filaments. The NiMn_2_O_4_-CF (NMO-CF) composite material was synthesized using a straightforward hydrothermal method. Initially, a total of 2.07 g of cobalt (II) nitrate hexahydrate (Mn(NO_3_)_2_.6H_2_O), 1.03 g of nickel(II) nitrate hexahydrate (Ni(NO_3_)_2_·6H_2_O), and 1.28 g of urea (CO(NH_2_)_2_) were dissolved in 30 mL of deionized water. The resulting mixture was then subjected to stirring for 20 min at a temperature of 60 °C. As a result of this process, a transparent pink solution was formed. Subsequently, the homogeneous precursor solution was carefully transferred into a Teflon-lined stainless-steel autoclave with a volume of 45 mL. The pre-treated carbon felt, measuring approximately 1 cm $\times$ 1 cm $\times$ 2 mm, was immersed in the solution and allowed to remain submerged for 3 h at different temperatures. After cooling to ambient temperature, the acquired specimen was subjected to a thorough rinsing with deionized water and subsequently subjected to a drying period of 3 h at a temperature of 80 °C. Ultimately, the specimens underwent annealing in a nitrogen (N_2_) environment at a temperature of 350 °C for 4 h.

### Electrode preparation

The electrochemical studies were utilized upon modified carbon felt with a 1 cm $\times$ 1 cm $\times$ 2 mm dimension. In order to evaluate the activity of the NMO-CF electrode towards the electrooxidation of ethylene glycol (EG), electrochemical investigations were carried out by cyclic voltammograms, chronoamperometry, and electrochemical impedance spectroscopy. 1.0 M NaOH aqueous electrolyte, a platinum wire as the counter electrode, and an Ag/AgCl (saturated KCl) as the reference electrode were employed in the three-electrode setup to conduct the necessary tests.

To conduct several electrochemical studies, such as cyclic voltammetry, chronoamperometry, and electrochemical impedance, an AUTOLAB workstation (PGSTAT128N) was used. The graphical user interface was created using Nova software (version 2.1). In order to measure the EIS experiment, an AC potential was applied that ranged from 0.1 Hz to 10^4^ Hz.

## Result and discussion

### Analysis

The chemical structure of the prepared spinel oxide and modified carbon felt composite were characterized using several surface and bulk analytical techniques. However, powder X-ray diffraction was employed to confirm the chemical structure of prepared spinel oxides.

**Figure 2:**
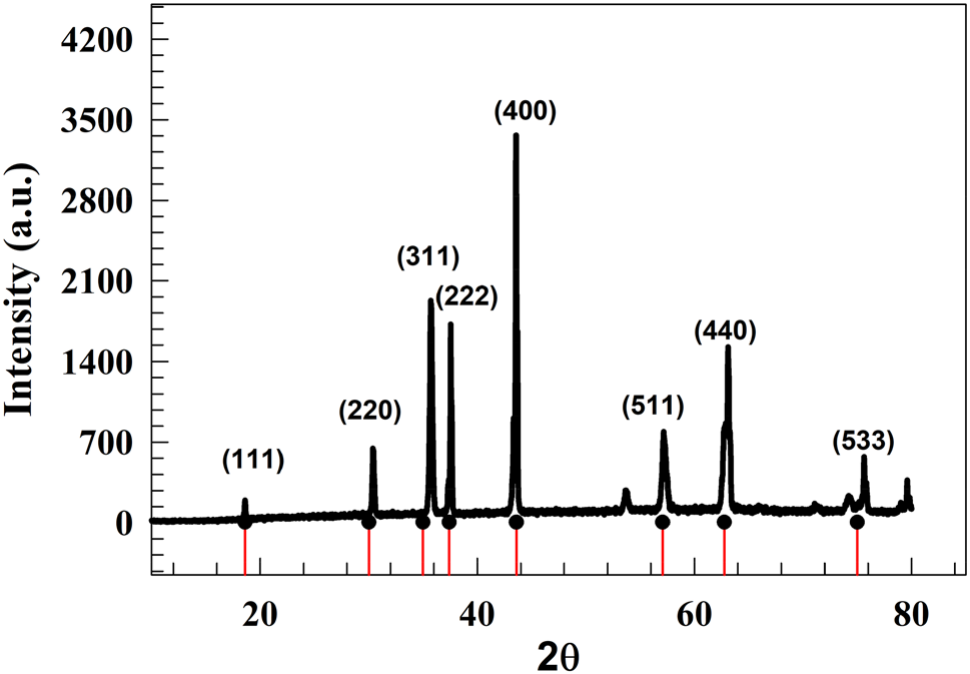
XRD chart of NiMn_2_O_4_.

Figure 2 depicts the composite NiMn_2_O_4_ spinel oxide. The diffraction peaks observed at angles of 18.6◦, 30.15◦, 35.71◦, 37.34◦, 43.6◦, 57.07◦, 62.78◦ and 57.8◦ can be attributed to the diffraction planes (111), (220), (311), (222), (400), (511), (440) and (533) of the spinel NiMn_2_O_4_, as depicted in Fig. 2. The observed peaks strongly correlate with the JCPDS No. 36–0083 and the findings described in the existing literature^37–40^.

**Figure 3:**
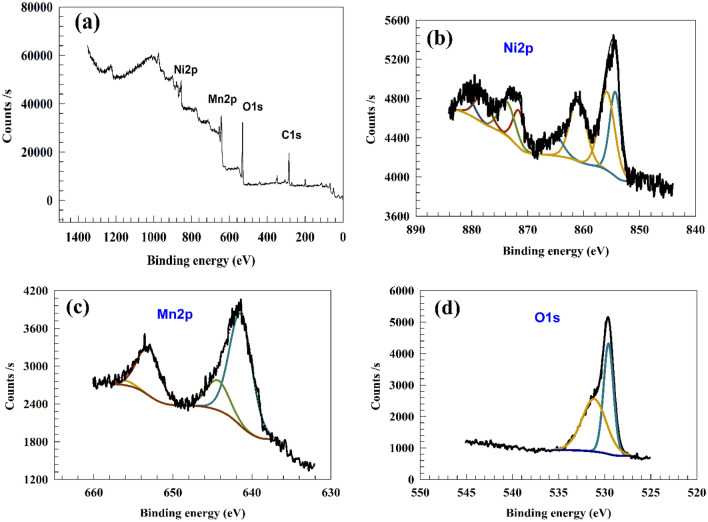
(**a**) survey of NiMn_2_O_4_, (**b**)Ni 2p, (**c**) Mn 2p, (**d**) O1s.

XPS analysis examined the surface atomic concentration and valence states of NiMn_2_O_4_ composites. Figure 3a displays the high-resolution XPS spectra of NiMn_2_O_4_ composites, which include the typical peaks for Mn (2p), Ni (2p), and O (1s) states. The X-ray photoelectron spectroscopy (XPS) analysis of nickel (Ni) revealed distinct peaks at energy levels of 854.6 eV and 871.7 eV, corresponding to the Ni (2p_3/2_) and Ni (2p_1/2_) electronic states, respectively. The observed discrepancy in binding energy, commonly referred to as spin-energy separation, which amounts to approximately 18 eV, provides evidence of Ni^2+^ ions within the NiMn_2_O_4_ composites^41–45^.

The Ni2p peaks observed at binding energy of 854.6 and 856.46 eV are corresponding to 2p_3/2_ Ni^2+^ and Ni^3+^ peaks^46,47^. The peaks seen at around 861.3 and 864.8 eV correspond to the satellite of Ni2p_3/2_. The peaks observed at 871.48, 874.34 eV, 878.6 and 881.03 eV correspond to the 2p_1/2_ state and the satellite of the Ni2p_1/2_ state, respectively^48–50^. Two supplementary peaks of shakeup satellites can be observed at energy levels of 860.8 eV and 878.7 eV, as depicted in Figure 3b.

However, the presence of two distinct peaks at 655.3 and 641.7 eV in Figure 3c can be attributed to the Mn2p_1/2_ orbital of Mn^2+^ ions and the Mn2p_3/2_ orbital of Mn^3+^ ions, respectively^51^.

The peaks observed at 644.7 and 655.3 eV in the spectrum correspond to Mn^3+^ ions, while the peak at 641.7 eV is attributed to Mn^2+^ ions^52^. The doublet Mn 2p, which exhibits a spin-orbit splitting of approximately 11.6 eV, is attributed to the presence of Mn^3+^. Figure 3d displays the deconvolution spectra for the O 1s, comprising three components. The lattice oxides (O^2−^) ions exhibited distinct peaks at 529.6 and 531.1 eV, which are typical of their composition.

**Figure 4:**
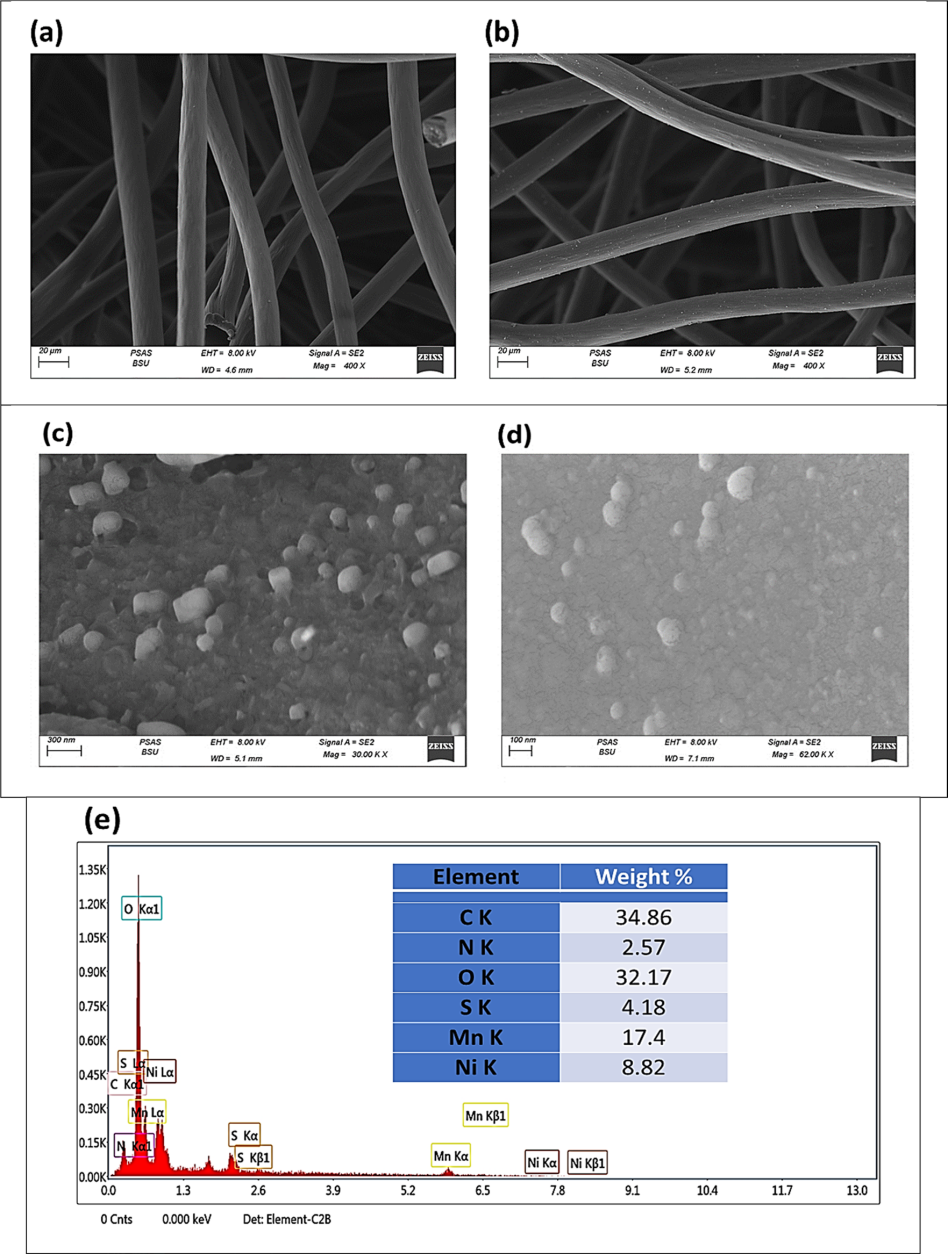
(**a**) Pristine CF, (**b**) NMO-CF, high magnification of NMO-CF, (**c**) before oxidation, (**d**) after oxidation, (**e**) EDX of NMO-CF.

The morphology of modified and unmodified were studied using high-resolution scanning electron microscopy (SEM). Figure 4a shows the unmodified carbon fiber felt in the nanoparticles' absence. Whereas the pretreatment of the carbon felt leads to the absence of contamination on an unmodified surface. The uniform distribution of active components on the CF fibers is investigated by examining scanning electron microscopy (SEM). Firstly, according to the data presented in Figure 4b, the surface of CF exhibits a uniform texture characterized by the presence of fine particles of NiMn_2_O_4_. As represented in Figure 4c, higher magnification of modified carbon felt showed a well-distribution of nanoparticles. Thus, nanoparticles with cubic structure shape were observed with a range of 75 ~ 120 nm.

The scanning electron microscopy (SEM) photographs obtained after 5 hours of electrolysis reveal a predominant similarity in the form of the NMO-CF electrode compared to its initial state before the prolonged operation (see Figure 4d). This observation confirms the remarkable stability exhibited by the suggested electrode material.

The use of energy-dispersive X-ray spectroscopy (EDX) is an essential methodology that facilitates the identification of the elemental ingredients of a provided surface. Moreover, it is employed to visually depict the spatial distribution of chemical components across the photographed area. The EDX obtained from the NMO-CF sample depicted in Figure 4e confirmed the presence of nickel (Ni), manganese (Mn), oxygen (O), nitrogen (N), sulfur (S), and carbon (C). Identifying components in the energy-dispersive X-ray (EDX) spectrum indicates the effective synthesis of the nickel manganese spinel oxide with a ratio between Ni and Mn of 1 to 2.

**Figure 5:**
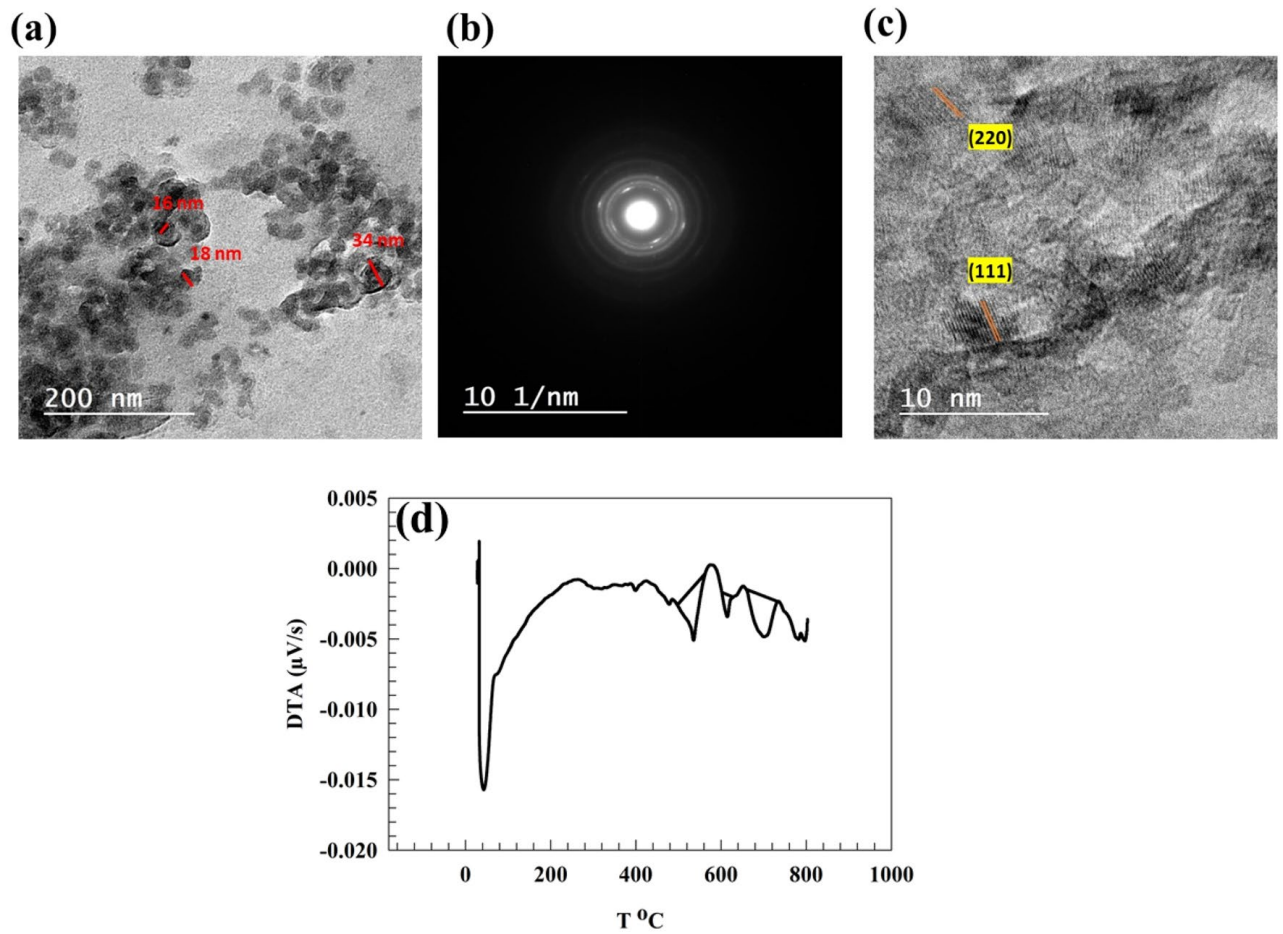
(**a**) TEM of NiMn_2_O_4_, (**b**) diffraction, (**c**) lattice spacing, (**d**) DTA of NiMn_2_O_4_-CF.

Furthermore, the morphological structure of NiMn_2_O_4_ was characterized by TEM. Figure 5a represents the particle size of 16 ~ 50 nm. Whereas, the cubic structure observed in XRD chart can be localized in TEM image. As represented in Figure 5b, the diffraction of NiMn_2_O_4_ confirm the presence of hkl of (111), (220), (400), and (440). Figure 5c shows lattice spacing of NiMn_2_O_4_ that the measured spacing corresponds to planes of (220), and (111) of NiMn_2_O_4_ crystals.

Figure 5d presents the differential thermal analysis (DTA) curves of the NMO-CF. As evident from the DTA curve, the initial breakdown stage commences at 519°C and concludes at 553°C with integration (-0.65 µV) attributed to the breakdown of the carbon felt. The second observed transition at 601 to 628 °C for integration of − 0.188 µV for carbon felt carbonization. The final stage was observed at 659 to 732, corresponding to converting residual oxide to spinel oxide with integration − 0.782 µV.

The surface and porosity of the pristine carbon felt, and modified NMO-CF were investigated using BET analysis. The analysis was performed by nitrogen adsorption without thermal correction at a pass temperature of 77.4 K and worm-free space of 10.3814 cm^3^.

A type III isotherm was observed, typical of microporous materials. Therefore, the BET surface area for unmodified and modified carbon felt was 13.28 and 328.24 m^2^/g, respectively. Moreover, the estimated adsorption average pore diameter for pristine CF and NMO-CF were 6.569 and 0.350 nm, respectively.

From BET analysis, our prepared electrocatalyst NiMn_2_O_4_/CF exhibited higher surface area (328.24 m^2^/g) over the other NiMn_2_O_4_ present in the literature. The surface area values for CuS/NiMn_2_O_4_ NCs^53^, NiMn_2_O_4_/CNF^54^, and NiMn_2_O_4_^55^, were found to be 54.83, 193, and 59.9 m^2^/g.

### Electrochemical investigation

#### Ethylene glycol oxidation

According to the oxidation mechanism of ethylene glycol compounds on Ni-based catalysts, NiOOH plays a catalytic function, and the oxidation steps takes place after Ni(OH)_2_ is converted to NiOOH^56,57^.

As a result, the following is the suggested mechanism on a nickel-based catalyst as follows^58^:

2 $${\text{Ni}}\left( {{\text{OH}}} \right)_{{2}} + {\text{O}}H^{ - } \leftrightarrow {\text{NiOOH}} + e^{ - }$$

3 $${\text{Ni}}^{{{2} + }} + {\text{HOCH}}_{{2}} {\text{CH}}_{{2}} {\text{OH}} \leftrightarrow {\text{Ni}}^{{{2} + }} \left( {{\text{HOCH}}_{{2}} {\text{CH}}_{{2}} {\text{OH}}} \right)_{{{\text{ads}}}}$$

4 $${\text{Ni}}^{{{2} + }} \left( {{\text{HOCH}}_{{2}} {\text{CH}}_{{2}} {\text{OH}}} \right)_{{{\text{ads}}}} + {\text{2 NiOOH}} \leftrightarrow {\text{Ni}}^{{{2} + }} \left( {{\text{HOCH}}_{{2}} {\text{CHO}}} \right)_{{{\text{ads}}}} + {\text{2 Ni}}\left( {{\text{OH}}} \right)_{{2}}$$

5 $${\text{Ni}}^{{{2} + }} \left( {{\text{HOCH}}_{{2}} {\text{CHO}}} \right)_{{{\text{ads}}}} + {\text{2 NiOOH}} + {\text{O}}H^{ - } \leftrightarrow {\text{Ni}}^{{{2} + }} ({\text{HOCH}}_{{2}} {\text{COO}}^{ - } )_{{{\text{ads}}}} + {\text{2 Ni}}\left( {{\text{OH}}} \right)_{{2}}$$

The electrode was first activated in KOH to create metal hydroxides and regenerate active species. The electrode was subjected to 50 cycles in a solution of 1.0 M NaOH at a scan rate of 20 mV s^−1^.

**Figure 6:**
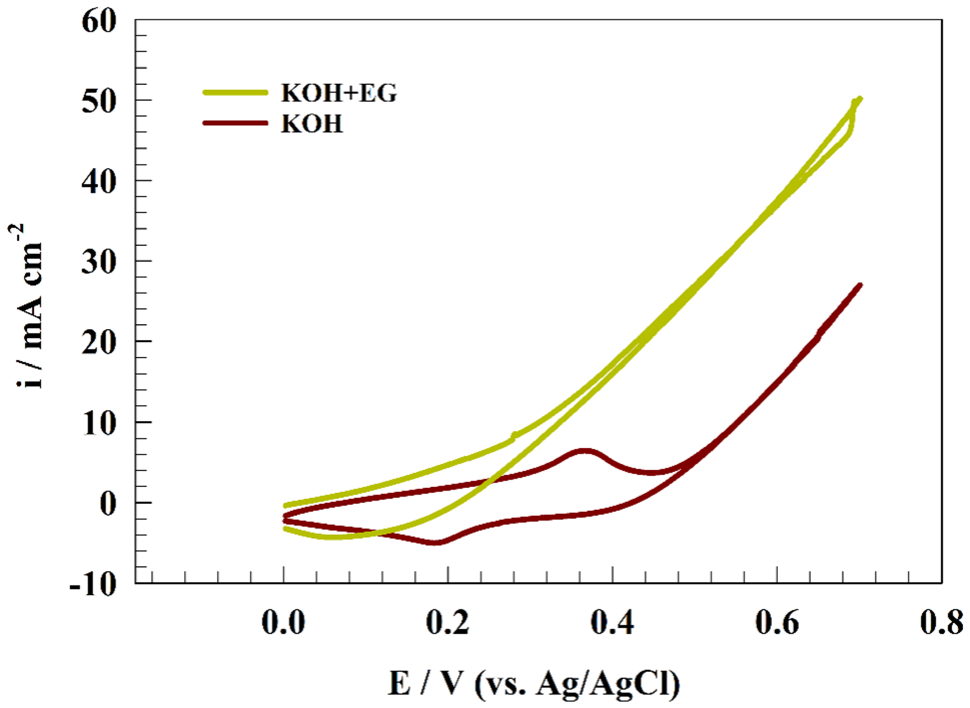
CV of modified NMO-CF electrode in the presence and absence of 1.0 M ethylene glycol.

Figure 6 displays the CVs of ethylene glycol electrochemical oxidation on the surface of NMO-CF in a solution of 1.0 M NaOH and 1.0 M fuels at a scan rate of 20 mV s^−1^. In the absence of ethylene glycol, only the redox peak observed attributed to the conversion of Ni(II)/Ni(III). Moreover, the addition of ethylene glycol led to the formation of distinctive oxidation peaks corresponding to the oxidation of ethylene glycol.

The carbon felt as a catalyst support has been extensively employed, particularly in cases where the catalyst exhibits restricted ethylene glycol oxidation activity. However, the application of CF has been found to enhance the electrode surface's electrical conductivity and improve the catalyst's stability.

The presence of active NiOOH species primarily influences ethylene glycol conversion. The calculation of surface coverage was performed using Eq. (6) in the absence of ethylene glycol for various scan rates ranging from 5 to 200 mV s^−1^. The equation provided can be estimated as follows^11^:

6 $${\text{I}}_{{\text{p}}} = \frac{{n^{2} F^{2} }}{4RT}\upnu \;{\text{A}}\;\Gamma$$

where Ip is current for EG oxidation, n is the number of consumed electrons, F is Faraday constant, A is electrode area, and Γ is surface coverage.

**Figure 7:**
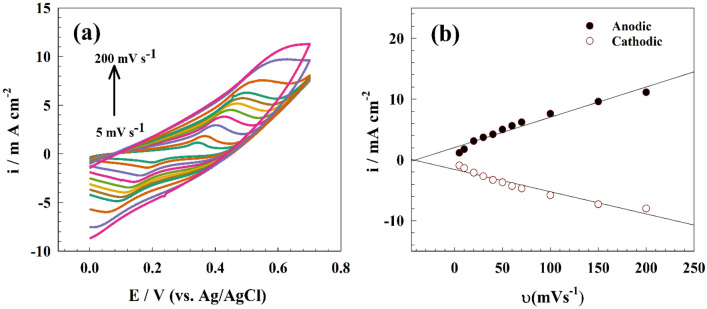
(**a**) CV of modified NMO-CF in 1.0 M NaOH at different scan rates, (**b**) linear relation between peak current versus of scan rate.

Figure 7a represents the CV of the modified NMO-CF electrode in 1.0 M NaOH in the absence of EG at a wide scan range. The surface coverage was established using a linear relation between oxidation current versus the scan rate (see Fig. 7b). Thus, the surface coverage provided for the modified surface was 7.66 $\times$ 10^−7^ cm s^−1^.

**Figure 8:**
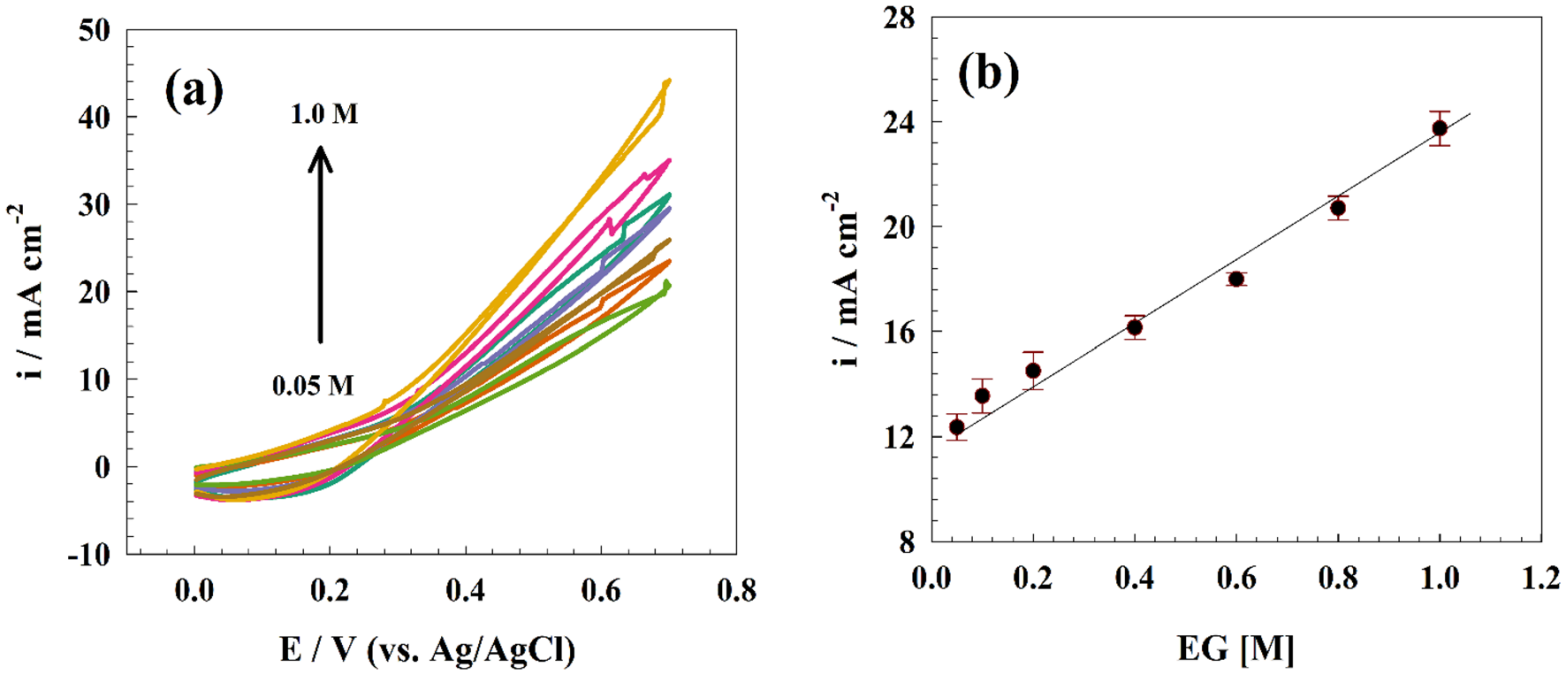
(**a**) CV of NMO-CF for different ethylene glycol concentrations, (**b**) linear relation between anodic current and ethylene glycol concentrations.

The investigation also encompassed the examination of the electrochemical reaction exhibited by the NMO-CF modified electrode in response to changes in the fuel concentration. The experiment involved altering the concentration of ethylene glycol within the solution, ranging from 0.05 M to 1.0 M. The solution also contained 1.0 M KOH; the scan rate used was 20 mV s^−1^, as depicted in Fig. 8a. The observed phenomenon indicates a positive correlation between the concentration of ethylene glycol and the magnitude of the anodic peak current. The data presented in this study suggests that the utilization of the proposed composite material shows promise for ethylene glycol electrooxidation in applications like fuel cells, and hydrogen production. It is worth noting that the effectiveness of this process is observed across a range of ethylene glycol concentrations. Figure 8b illustrates the correlation between the concentration of ethylene glycol and the magnitude of the anodic peak current.

**Figure 9:**
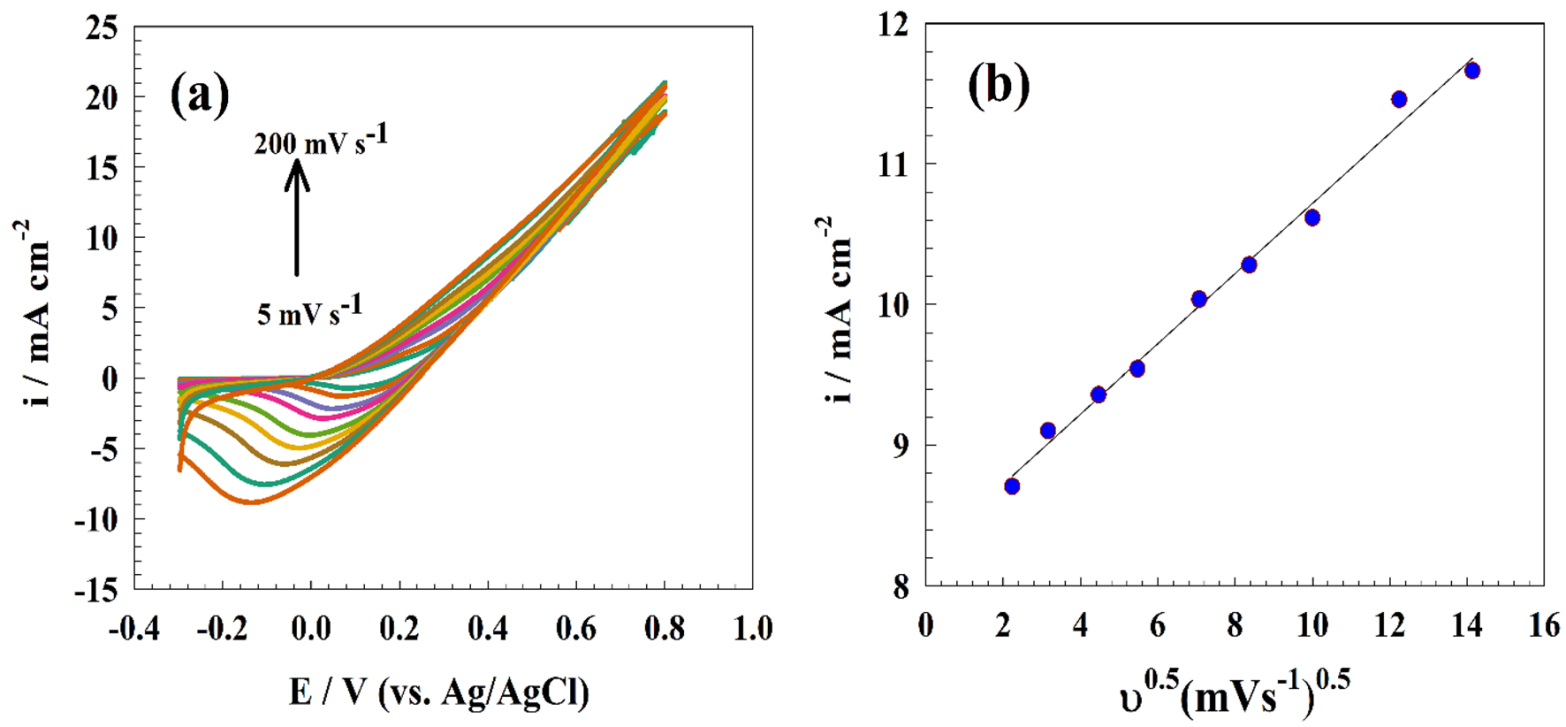
CVs of NMO-CF in ethylene glycol and NaOH at different scan rates, (**b**) Linear relation between anodic current versus square root of scan rate.

For additional electrocatalyst experiments, cyclic voltammetry was used to examine the effects of various scan rate ranges of (5–200 mV s^−1^) in the solution of 1.0 M ethylene glycol and 1.0 M NaOH, as shown in Fig. 9a.

The linear relationship created by graphing the anodic peaks current against the square root of scan rates is shown in Fig. 9b, The ethylene glycol oxidation process on the pre-prepared electrocatalysts is diffusion-controlled, and the diffusion coefficient was calculated using the following Randles–Sevcik equation based on the slope of the Ip vs. ν^1/2^ linear relation^59,60^:

7 $${\text{I}}_{{\text{p}}} = 2.99 \times 10^{5} {\text{n }}\left( {1 - \upalpha } \right){\text{n}}_{{\text{o}}} {\text{AC}}_{{\text{o}}} {\text{D}}^{0.5}\upupsilon ^{0.5}$$

The maximum oxidation current is Ip, the anodic charge transfer coefficient is α, ʋ is the scan rate (V s^−1^), C_o_ is the initial ethylene glycol concentration (mol cm^3^), and D is the diffusion coefficient (mol cm^−1^) and A the electrode's surface area. The diffusion coefficient estimated for modified NMO-CF as 1.6 $\times$ 10^−7^ mol cm^−1^. However, the small diffusion coefficient value for ethylene glycol toward the NMO-CF electrode can be examined by high ethylene glycol viscosity that limits the movement of fuel molecules through the carbon felt electrode. The difficulties of ethylene glycol diffusion can also be observed by slight changes in the anodic current density with the increased scan rate, as indicated in Fig. 9a.

**Figure 10:**
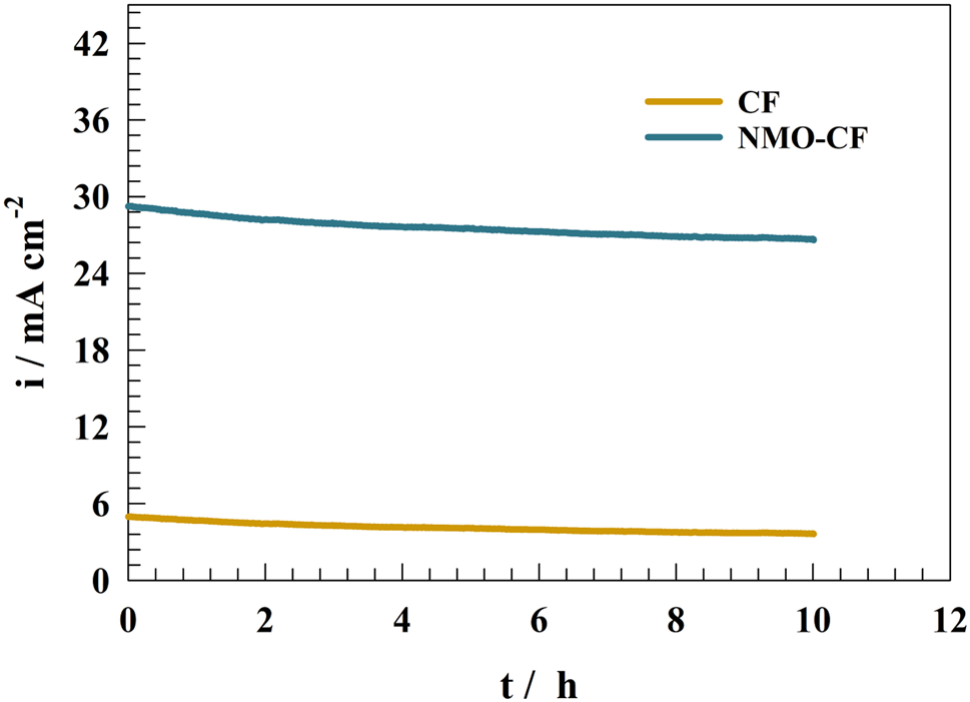
Long-term stability of ethylene glycol after 10 h of oxidation.

The endurance of NMO-CF and CF were assessed by observing the constant potential chronoamperometry over a period of 10 h, while maintaining a constant potential of 0.55 V (vs. Ag/AgCl) (see Fig. 10). In case of NMO-CF, a high stability can be noticed for ethylene glycol oxidation. Whereas, the current decreased by 9.8% of the initial value after 10 h of continuous oxidation. The decrement current can be explained by accumulation of residual carbon materials or formation of nickel/manganese carbonate after adsorption of CO as a result of oxidation process. Furthermore, mechanical corrosion of electrode by gas evolution after ethylene glycol conversion. Therefore, presence of NMO nanoparticles improved the stability of CF towards the ethylene glycol oxidation.

#### Hydrogen evolution

**Figure 11:**
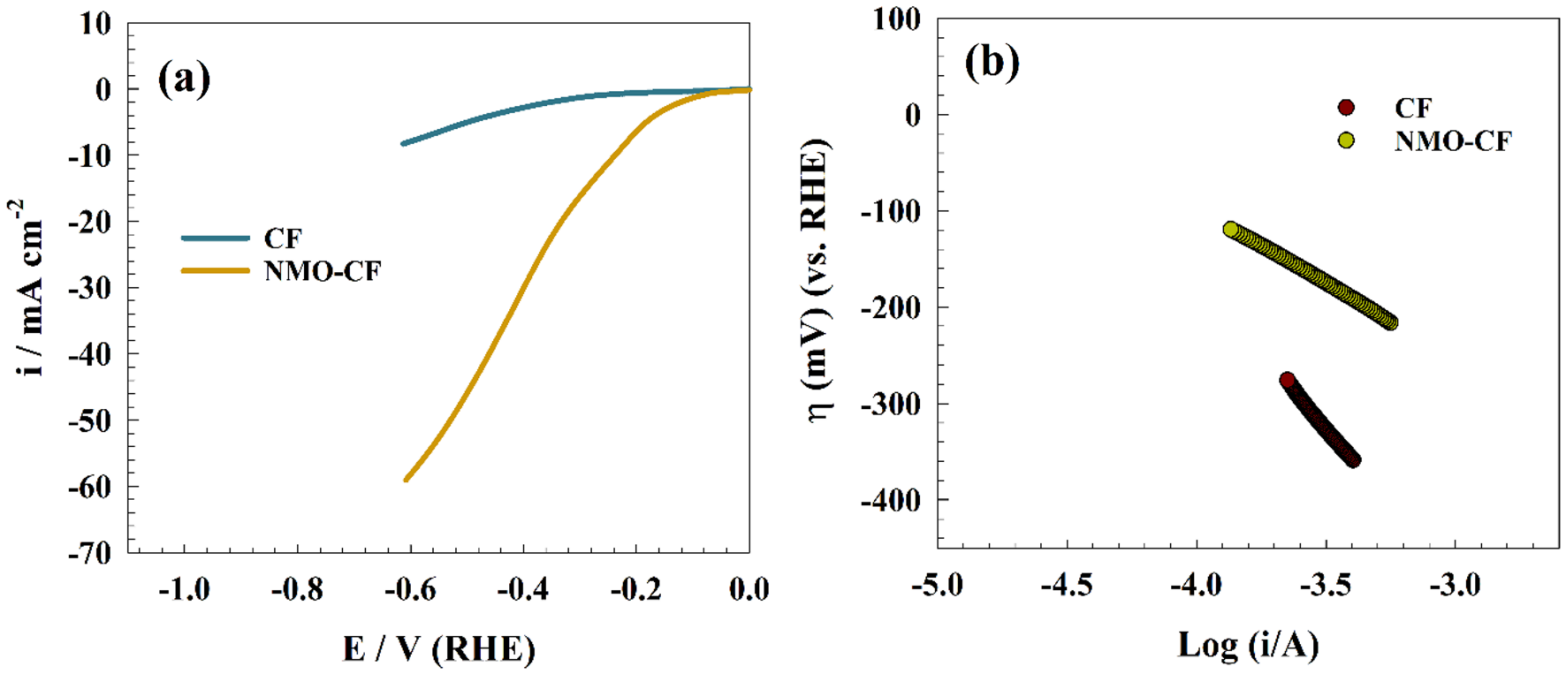
Linear sweep voltammetry of modified surfaces for HER., (**b**) Tafel plot of hydrogen evolution upon CF an NMO-CF electrodes.

The second aim of this study was to examine the HER on a surface that has undergone modification using NMO-CF. Figure 11a illustrates the linear sweep voltammetry of the NMO-CF surface in alkaline medium. The merging of carbon support with spinel oxides led to a discernible enrichment in current density for the improved NMO-CF. The alteration resulted in enhancements in both the electrical and adsorption properties. The hydrogen evolution reaction (HER) can be mathematically described in an alkaline environment using the following steps: The Volmer step, also referred to as the first stage of HER, involves the adsorption of hydrogen ions onto the surface of the electrode. In the subsequent phase, also known as the Tafel reaction, two adsorbed hydrogen ions on the surface combine. Alternately, the phenomenon may involve the Heyrovsky mechanism, which involves the formation of a direct bond between a hydrated proton in the medium and a surface-adsorbed hydrogen atom.

The application of the Tafel polarization curve by using LSV facilitates the determination of the rate-controlling step, specifically either the first or subsequent step, in HER. The Tafel plot depicted in Fig. 11b showcases the performance of the NMO-CF electrode in the context of HER. The Tafel slopes of modified an unmodified carbon felt, revealing that NMO-CF exhibits a Tafel slope of 98 mV dec^−1^, whereas untreated carbon felt has a Tafel slope of 138 mV dec^−1^. The Tafel slope value for the NMO-CF was consistent with previous modified surface values published for the Ag@CNT/glassy carbon (148 mV dec^−1^)^61^ and Ni_2_Fe@porous carbon (83 mV dec^−1^)^62^.

**Figure 12:**
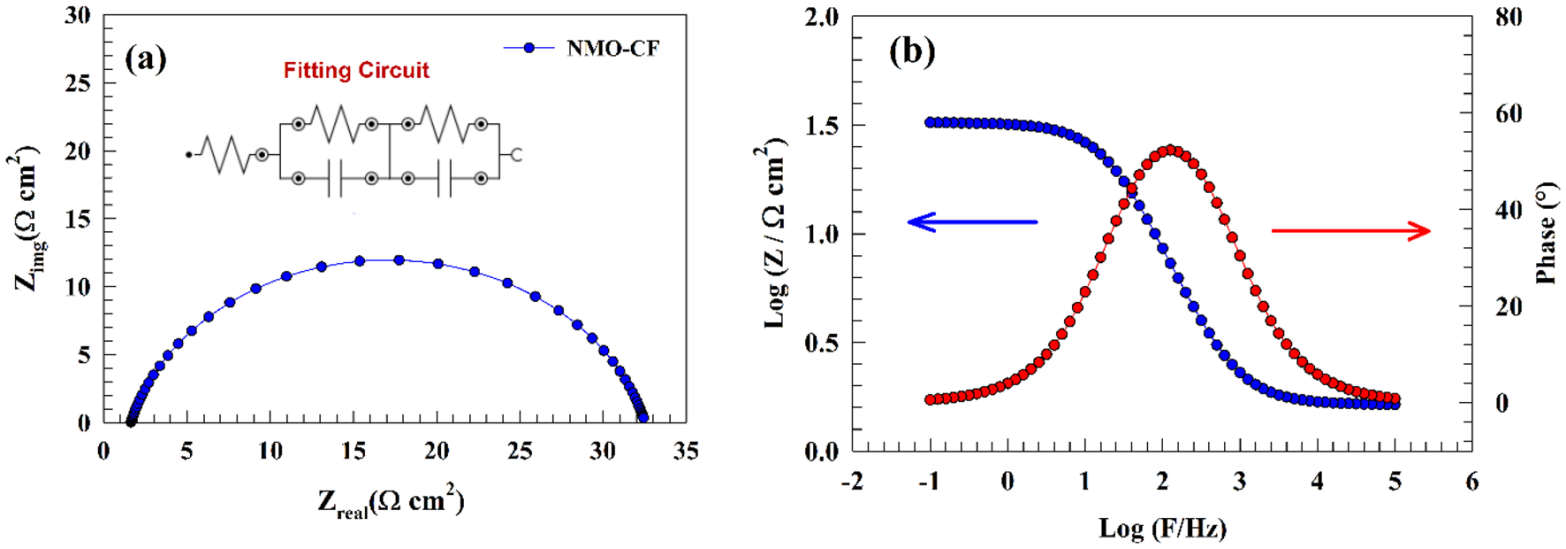
EIS representation of NMO-CF (**a**) Nyquist, (**b**) Bode plots.

The utilization of electrochemical impedance spectroscopy (EIS) was employed to examine the HER of the modified NMO-CF electrodes. Figure 12a demonstrates the use of HER through the utilization of electrochemical impedance spectroscopy (EIS) while subjecting it to a consistent alternating current (AC) potential of − 0.45 V relative to the reversible hydrogen electrode (RHE). The magnitudes of resistance exhibited variability; nevertheless, the Nyquist plot created by the modified electrode NMO-CF consistently revealed semi-circular patterns. The electrochemical manufacturing process can be described as a phenomenon involving the transfer of charges, as supported by the data acquired using electrochemical impedance spectroscopy (EIS). The EIS data was analyzed by employing the NOVA software for fitting purposes. The NMO-CF electrode demonstrated a dual circuit topology, where one cell was connected to the solution resistance. The capacitor and resistance were connected in parallel within the cell, as seen in the inset of Fig. 12a.

In addition, the presence of a constant phase component signifies the presence of surface heterogeneity, which has implications for the effectiveness of hydrogen production. The measured charge transfer resistance of the NMO-CF surface is determined to be 33 Ω cm^−2^. A favorable link has been seen between a reduction in resistance value and an augmentation in electrode activity. The estimated parameters of the electrochemical impedance spectroscopy (EIS) results are presented in Table 1. The properties of the modified electrodes NMO-CF when exposed to a potential of − 0.45 V in a 1.0 M NaOH solution are depicted in Fig. 12b, which showcases the bode graphs. Whereas, the pure charge transfer process can be observed. The associated Bode plot shows how the nanostructured thin films act as electrocatalysts. According to the Bode figure, the phase angles at the highest frequency (f_max_) exhibit the following conductive trend at phase angle 53°.

**Table 1:** EIS parameters for NMO-CF electrode for HER.

Electrode	Rs (Ω cm^−2^)	R_1_ (Ω cm^−2^)	C1 (F)	R_2_ (Ω cm^−2^)	C2 (F)
NMO-CF	2.15	3.21	0.0000173	33.74	0.000308

## Conclusion

The nickel manganese loaded to carbon felt was successfully prepared using the hydrothermal method. Whereas the structure of the prepared materials was characterized using different analytical techniques.

The electrode showed high activity toward ethylene glycol oxidation and hydrogen production in an alkaline medium. The diffusion of ethylene glycol toward the NMO-CF electrode was observed to be slightly small due to the interaction between EG and water. Furthermore, the electrode showed noticeable stability after 10 h of continuous oxidation. Thus, the electrode's durability is verified by structure stability by scanning electron microscopy. On the other hand, the NMO-CF reached a high current density (50 mA cm^−2^) for hydrogen production at an overpotential of 480 mV (vs. RHE).

## Data Availability

The datasets used and/or analysed during the current study are available from the corresponding author on reasonable request.
